# 

**Published:** 1887-04

**Authors:** 


Whole Number, 328. APRIL, 1887. Vol. LIV, Number 4.

LIBRARY
INDEXED
S. G. O.

THE

### CHICAGO MEDICAL JOURNAL AND EXAMINER

Editor: S. J. JONES, M.D., LL.D.

PUBLISHED MONTHLY BY

THE CLARK & LONGLEY COMPANY,

Under direction of

THE CHICAGO MEDICAL PRESS ASSOCIATION,

308-316 DEARBORN STREET.

